# Composite Augmentation and Feature Integration Reconstruction for Occluded Person Re-Identification

**DOI:** 10.3390/s26103114

**Published:** 2026-05-15

**Authors:** Hao Tie, Chentao Hu, Yibo Chen, Lingbing Tao, Yanbing Chen

**Affiliations:** 1 Keyi College, Zhejiang Sci-Tech University, Shaoxing 312369, China; th18220117@ky.zstu.edu.cn; 2 School of Computer Science and Technology, Zhejiang Sci-Tech University, Hangzhou 310018, China; 202230704060@mails.zstu.edu.cn (C.H.); 2024220603012@mails.zstu.edu.cn (Y.C.); lb_tao@zstu.edu.cn (L.T.)

**Keywords:** occluded person re-id, vision transformer, data augmentation, feature interaction, feature reconstruction

## Abstract

Most existing occluded person re-identification (Re-ID) methods rely on external cues to separate the visible parts of the person from the occluded regions, aiming to achieve alignment based on visible body parts. However, such methods often overlook the complexity of different occlusion scenarios. Moreover, there exists a severe imbalance between the training and testing sets in current occlusion datasets, which further undermines the model’s generalization ability. In this paper, we propose a method named Composite Augmentation and Feature Integration Reconstruction For Occluded Person Re-Identification. Firstly, a Composite Data Augmentation Module is designed to increase the number of occluded samples in the training set, thus alleviating data imbalance. Next, a feature interaction module is introduced to perform bidirectional interactions between global and local features, which helps reduce the impact of occlusion-induced noise and feature redundancy in complex scenes, thereby improving feature representation efficiency. Finally, a Feature Reconstruction Module retrieves the top-k nearest neighbor images from the gallery to reconstruct the occluded body parts, enabling more complete person matching. Experimental results on five challenging occlusion datasets demonstrate that the proposed method achieves superior performance.

## 1. Introduction

Person re-identification (re-id) aims to match individuals across images from non-overlapping cameras. While deep learning has advanced re-id, occlusion remains a major challenge. Objects like umbrellas, signs, vehicles (Object-to-Person, OTP), or other people (Person-to-Person, PTP) often obscure parts of the target’s body, complicating feature extraction from occluded images. Despite progress, existing methods struggle with large-area occlusions or those caused by various objects, prompting significant research in occluded person re-identification.

**Figure 1:**
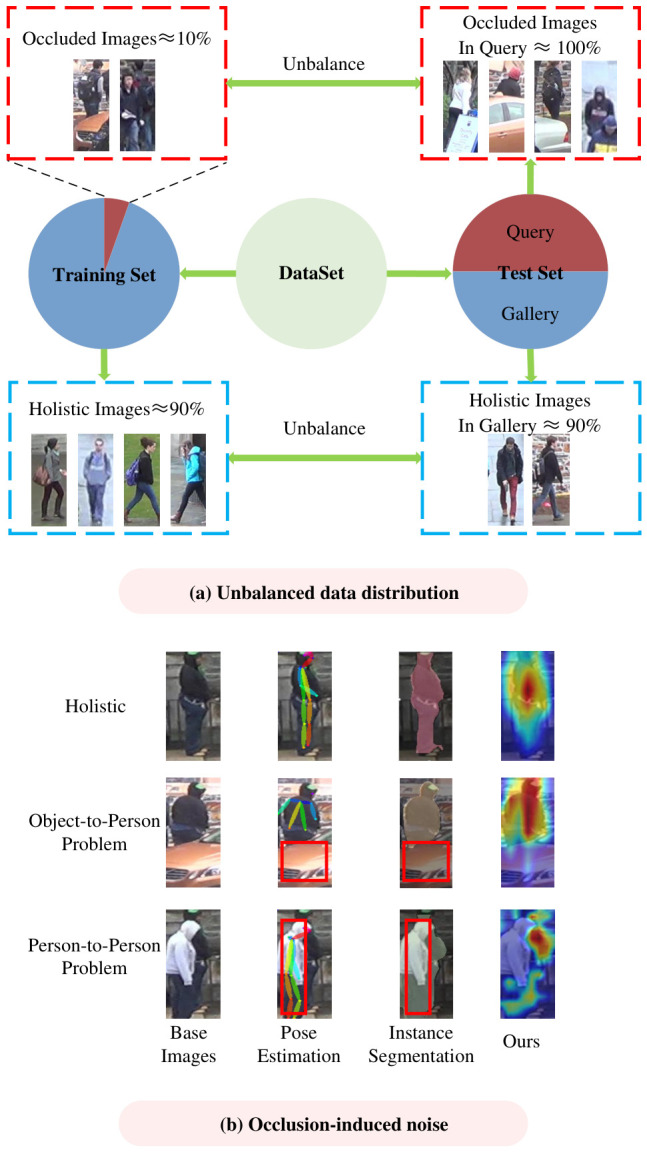
(**a**) The challenge in occluded person re-identification lies in the unbalance between occluded and non-occluded images, with 90% of training images being non-occluded, while most query images are occluded. (**b**) The OTP and PTP occlusion types are illustrated by comparing holistic and occluded images, highlighting the challenges caused by different occlusion types in person re-identification tasks.

Occluded person re-identification faces two main challenges: (1) Unbalanced data distribution: As shown in Figure 1a, the training set is mostly composed of holistic person images, while the query set in the test set contains predominantly occluded person images, and the gallery set is largely made up of holistic images. This imbalance increases the difficulty of occluded person re-identification. (2) Occlusion-induced noise: Unlike traditional re-identification, which assumes full visibility, occluded person re-identification deals with noise from occlusions that obscure critical features, making feature extraction and matching more challenging.

To address the issue of unbalanced data distribution, current methods typically employ data augmentation techniques to enhance data diversity [1,2]. However, traditional data augmentation strategies, such as randomization processing or direct occlusion addition to images, have multiple limitations. Firstly, these methods may inadvertently alter the crucial details of key human body parts, thereby reducing the accuracy of person recognition. Secondly, the traditional data augmentation process usually acts on the same sample in a continuous and singular manner at the data level. That is, only the same augmentation logic is applied to individual samples, resulting in a high degree of correlation among augmented samples. This significantly impairs the model’s generalization ability.

Regarding the challenge of occlusion noise in pedestrian feature extraction, most solutions attempt to distinguish between the visible and occluded parts by utilizing external clues such as pose estimation and human parsing [3,4,5]. However, these methods face two major issues in practical applications. Firstly, in the case of the OTP occlusion, as depicted in Figure 1b, although external cues can capture the information of the visible parts of pedestrians, the presence of object occlusion will disrupt the continuity and integrity of pedestrian features. As a result, it is difficult to obtain the features of the occluded areas, making it challenging for the model to construct a complete representation of pedestrian features. Secondly, in the scenario of the PTP occlusion, as shown in Figure 1b, due to the domain gap between the pre-trained dataset and the target occluded images, external models struggle with generalization. This may lead to the mixing of features between the target pedestrian and the occluding pedestrian.

To address the above issues, we propose the Composite Augmentation and Feature Interaction Reconstruction (CAFIR) method. Different from existing methods that heavily rely on external cues (e.g., pose estimation, human parsing) [3,4,5], our CAFIR provides a novel solution without requiring additional external models. The model consists of three modules: the Composite Data Augmentation Module (CAM), the Feature Integration Module (FIM), and the Feature Reconstruction Module (FRM).

The motivation behind our proposed CAFIR stems from three key observations:

- **Data imbalance challenge:** Existing occlusion datasets suffer from a severe imbalance between training and testing sets. As illustrated in Figure 1a, approximately 90% of training images are non-occluded holistic images, while most query images in the test set are occluded. This distribution mismatch significantly undermines model generalization ability. Traditional data augmentation methods often apply single augmentation logic to individual samples, leading to high correlation among augmented samples and limiting diversity.
- **Limitations of external cue-based methods:** While methods relying on external cues (pose estimation, human parsing) can capture visible parts, they face two critical issues: (1) For OTP occlusion, object presence disrupts feature continuity, making it difficult to reconstruct occluded areas; (2) For PTP occlusion, domain gaps between pre-trained models and target images lead to feature mixing between target and occluding pedestrians.
- **Need for holistic feature interaction:** Shallow global and local features alone may overlook important person details. A bidirectional interaction mechanism between global and local features can effectively reduce occlusion noise and feature redundancy.

The CAM module adopts a three-branch input strategy, where three augmented inputs and one base input are paired to form triplets. The base input, combined with an augmented branch based on prior knowledge, serves as the main branch to generate composite samples with diverse occlusion types, addressing the imbalance between occluded and non-occluded samples. Additionally, by combining holistic images with occluded ones, it avoids the homogenization of augmented samples. The FIM and FRM modules complement each other within the model. The FIM module employs a global–local feature bidirectional interaction strategy, aiming to reduce occlusion noise and feature redundancy caused by other pedestrians, while refining key information in the image. This helps the model focus on more discriminative features, thereby enhancing its generalization ability. The FRM module, based on the generalized cue information, selects complete pedestrian images from a gallery to fill in the missing parts of occluded images, effectively solving the problem of discontinuity and incompleteness in occluded images.

The main contributions are outlined below:

(1) We propose a novel Composite Data Augmentation Module (CAM) that adopts a three-branch input strategy to generate diverse occlusion types and effectively address the data imbalance between occluded and non-occluded images in the training dataset. This module provides a simpler and more effective data augmentation strategy for occluded person re-identification, effectively mitigating the homogenization problem caused by single-sample augmentation.

(2) We present a feature interaction module (FIM) with a global–local feature bidirectional interaction strategy. By enhancing the information exchange between global and local features, it effectively reduces occlusion noise caused by objects and redundancy introduced by other pedestrians, while refining key information in the image. This guides the model to focus on more discriminative regions and learn cue information with enhanced generalization.

(3) We introduce a Feature Reconstruction Module (FRM) that leverages the top-k nearest neighbor images from the gallery to reconstruct occluded body parts, solving the problem of discontinuity and incompleteness in occluded images through visibility-score guided reconstruction.

(4) Extensive experiments indicate that our proposed model achieves superior performance compared to current methods on three occluded datasets and two partial datasets. Furthermore, its performance is comparable to previous methods on two holistic datasets.

## 2. Related Work

### 2.1. Occluded Person Re-ID

In the task of occluded person re-identification, matching query images in occlusion scenarios with holistic gallery images is a significant challenge. This task requires not only the precise extraction of fine-grained features but also the effective handling of large intra-class variations. Real-world scenarios are inherently complex and uncertain, with surveillance images subject to various internal factors, such as changes in posture and clothing, as well as external factors like viewpoint changes, occlusion by other objects or non-target persons, etc.

Recently, Ning et al. [6] provided a comprehensive survey on occluded person re-identification, summarizing existing techniques and discussing future directions. To address occlusion issues, there are two mainstream approaches: one based on external cues and the other on rigid stripe partitioning [7]. The methods based on external cues aids in feature extraction by introducing external models, such as pose estimation and human parsing. For example, Miao et al. [5] used a human pose estimator to generate semantic keypoints of the human body, expanding high-order semantic features to effectively align the visible parts of persons. Wang et al. [8] proposed a pose-guided feature disentanglement method that leverages pose information to separate visible and occluded regions. Cioppa et al. [9] employed a human parsing model trained on the COCO dataset [10] to generate pseudo-pixel labels for different parts of person images and focused on the most representative parts using a part attention module to achieve precise part-to-part matching.

The other methods use rigid stripe partitioning, where images or feature maps are divided into vertical or horizontal segments. The Part-based Convolutional Baseline (PCB) network proposed by Sun et al. [7] provides a strong Baseline for person re-identification. This method enhances the consistency of local features by uniformly dividing them using Refined Part Pooling (RPP). Additionally, Yan et al. [11] proposed a partitioning strategy based on the information characteristics of different human body parts, enabling the model to learn more stable feature representations in occluded and noisy feature maps.

### 2.2. Person Re-ID Based on Data Augmentation

Data augmentation is a direct and effective approach to improving the performance of occluded person re-identification, as demonstrated in several studies [1,2,12,13]. The core advantage of data augmentation lies in its ability to significantly increase the number of training images while enriching their diversity. Generally, data augmentation methods are typically classified into two primary types:

Pixel-level operations: These methods manipulate the pixels of an image. Typically, a rectangular region is selected within an image, and its pixels are replaced with random values or background noise. Yan et al. [1] employed two data augmentation techniques in their study: Random Erasing (RE) [2] and Batch-constant Erasing (BcE) [1]. Random Erasing removes small random regions from the image, encouraging the model to learn to ignore irrelevant information or occluded parts, thus enhancing robustness. On the other hand, Batch-constant Erasing removes larger and continuous areas.

Occluding object fusion strategies: These methods involve constructing a container to store occluding objects that commonly appear in the dataset. A selected image or feature is then combined with the occluding objects, which are applied to the image or feature and passed through the network for training. For instance, Jia et al. [12] proposed an Occlusion Sample Augmentation (OSA) strategy, which randomly selects predefined obstacles to cover person images for data augmentation. However, a limitation of this approach is that it requires manual cropping and pasting of occluding objects, which is relatively cumbersome. Additionally, Wang et al. [13] introduced a Feature Diffusion Module that simulates the fusion of multiple person features through cross-attention operations in a memory bank. However, this method has its drawbacks, as it requires additional storage space to save person representations.

More recently, Liu et al. [14] proposed joint augmentation and part learning for unsupervised clothing change person re-identification, which addresses the challenge of person re-identification across clothing changes. This work is highly relevant to our research on occlusion handling.

### 2.3. Person Re-ID Based on Transformer

In recent years, Vision Transformers (ViTs) [15] achieve remarkable results in various visual tasks such as image classification, image segmentation, and visual question answering. He et al. [16] are the first to introduce Vision Transformers into the field of person re-identification, proposing the TransReID model, which is the first person re-identification model fully based on the Vision Transformer architecture, completely replacing the traditional Convolutional Neural Networks (CNNs) [17] for feature extraction. This shift not only expands the design space for person re-identification models but also demonstrates the potential of Transformer architectures in complex visual tasks, overcoming CNNs’ limitations in capturing long-range dependencies due to their limited receptive fields.

Following this, numerous researchers explore further applications and innovations based on TransReID. Among them, Wang et al. [8] introduce a pose-guided matching and distribution mechanism with the Transformer as the backbone. This mechanism accurately identifies visible parts of a person and enhances their feature representation by incorporating semantic views. Tan et al. [18] also use Vision Transformers as the backbone and propose a Dynamic Prototype Masking (DPM) strategy. This strategy precisely focuses on critical image parts using dynamic masks, significantly improving the model’s ability to handle occluded person re-identification tasks without additional models. Xu et al. [19] utilize graph matching techniques to focus on visible regions within image groups, cleverly leveraging Transformers to construct a nearest-neighbor feature set for effective information recovery from holistic data. Yue et al. [20] proposed a local correlation ensemble with graph convolutional network based on attention features for cross-domain person re-identification. More recently, Guo et al. [21] proposed the Feature Completion Transformer, which achieves state-of-the-art performance on multiple occluded person re-identification benchmarks. Additionally, Wu et al. [22] proposed extended cross-modality united learning for unsupervised visible-infrared person re-identification, further advancing the field. Yue et al. [23] also proposed PGAN, a part-based non-direct coupling embedded GAN for person re-identification.

## 3. Methodology

**Figure 2:**
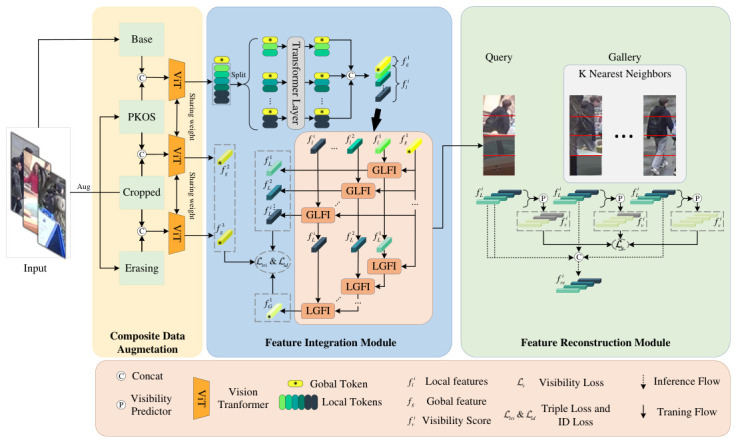
The overall framework of the CAFIR framework, which includes the Composite Data Augmentation Module, the feature interaction module, and the Feature Reconstruction Module.

In this section, we introduce the CAFIR framework, shown in Figure 2, which consists of three main modules: the Composite Data Augmentation Module, the feature interaction module, and the Feature Reconstruction Module. The Composite Data Augmentation Module concatenates three augmented data samples with an original input to form a triplet data format, thereby enhancing the learning of person representations. The feature interaction module sequentially enhances local and global features, obtaining context-related features of the person’s global features and holistic correlation features of local features through global–local interactions. This approach strengthens the detailed features of persons while suppressing noise from partial occlusions. The Feature Reconstruction Module leverages the top *k* nearest neighbor images in the gallery to reconstruct the occluded features of the person. The Feature Reconstruction Module, building on feature interaction, uses the most similar *k*-nearest neighbor images from the gallery to reconstruct the occluded features.

### 3.1. Composite Data Augmentation

Data augmentation plays a pivotal role in occluded person re-identification by expanding both the size and diversity of the dataset. By generating multiple image samples under various occlusion conditions. However, it is essential to carefully evaluate the potential impact of augmentation on the integrity of critical image details to ensure the preservation of data quality and stability. This section presents a comprehensive data augmentation strategy that aims to balance the augmentation of data diversity with the retention of image detail fidelity.

We adopt Vision Transformer (ViT) [16] as the backbone network. We assume a person image $I\in R^{H\times W\times C}$, where *H*, *W*, and *C* represent the height, width, and number of channels of the image, respectively. The initial image undergoes several augmentation steps. Base augmentation is applied to obtain $I_{\mathrm{base}}=BA\left(I\right)$. Center cropping is performed to produce $I_{\mathrm{cropped}}=CA\left(I\right)$. Regional Random Erasing is applied to generate $I_{\mathrm{erased}}=EA\left(I\right)$. Prior-knowledge-based augmentation [24] is employed to yield $I_{\mathrm{PKOS}}=PA\left(I\right)$. BA(·), CA(·), EA(·), and PA(·) represent base augmentation, cropping augmentation, erasing augmentation, and prior-knowledge-based augmentation, respectively. These four augmented images are concatenated in pairs to form feature map triplets $\left[I_{1},I_{2},I_{3}\right]$. The expression is as follows:

(1) $$\begin{array}{cc} I_{1} & =\mathrm{concat}(I_{\mathrm{base}},I_{\mathrm{PKOS}}),\\ I_{2} & =\mathrm{concat}(I_{\mathrm{cropped}},I_{\mathrm{PKOS}}),\\ I_{3} & =\mathrm{concat}(I_{\mathrm{cropped}},I_{\mathrm{erased}}), \end{array}$$

where concat(·,·) represents the concatenation operation. Subsequently, ViT is employed as the backbone network to extract features, and the image triplet is sent to a parameter-shared multi-branch backbone network. The image $I_{1}$ is divided into *N* patches $\left\{I_{p}^{i}|i=1,2,\ldots ,N\right\}$ using a sliding window approach. This can be expressed as:

(2) $$N=N_{H}\times N_{W}=\left\lfloor \frac{H+S-P}{S}\right\rfloor \times \left\lfloor \frac{W+S-P}{S}\right\rfloor ,$$

where *S* represents the stride, *P* represents the size of each patch, and $\left\lfloor \cdot \right\rfloor$ denotes the floor function. $N_{H}$ and $N_{W}$, respectively, represent the number of patches in the vertical and horizontal directions after splitting. Following linear projection, a learnable class token $I_{\mathrm{cls}}$ is prepended to aggregate information from the image patches. Before feeding into the Transformer blocks, learnable positional embeddings $E_{p}\in R^{(N+1)\times D}$ are added to the patch embeddings to preserve positional information, where *D* is the channel dimension. This can be mathematically expressed as:

(3) $$Z_{0}=\left[I_{\mathrm{cls}};\;F\left(I_{p}^{1}\right);F\left(I_{p}^{2}\right);\cdots ;F\left(I_{p}^{N}\right)\right]+E_{p},$$

where:

- $Z_{0}\in R^{(N+1)\times D}$ represents the initial input to the Transformer encoder, consisting of the class token and *N* patch embeddings.
- F(·) denotes the linear projection operation that maps each patch to a *D*-dimensional embedding space.
- $E_{p}\in R^{(N+1)\times D}$ represents the learnable positional embeddings added to preserve spatial information.
- *N* denotes the total number of image patches.
- *D* represents the channel dimension of the embeddings.
- $f_{cls}\in R^{1\times D}$ represents the class token of the input image, which aggregates information from all patches.
- $f_{p}^{N}\in R^{1\times D}$ corresponds to the patch embeddings for each patch after processing through the Transformer.

Based on this, $Z_{0}$ passes through *l* Transformer layers to obtain the output, $Z_{l}=\left[f_{cls};f_{p}^{1},f_{p}^{2},\cdots ,f_{p}^{N}\right]$, $Z_{l}\in R^{(N+1)\times D}$. $f_{cls}\in R^{1\times D}$ represents the class token of the input image, and $f_{p}^{N}\in R^{1\times D}$ corresponds to the patch embeddings for each patch. By passing $f_{cls}$ through a shared Transformer layer, the final global feature representation $f_{g}^{1}\in R^{1\times D}$ is obtained for $I_{1}$. The global feature representations $f_{g}^{2}\in R^{1\times D}$ and $f_{g}^{3}\in R^{1\times D}$ are extracted from $I_{2}$ and $I_{3}$ using ViT.

### 3.2. Feature Interaction Module

The relationships between local human body features and long-range dependencies are crucial for constructing robust person representations. However, many existing methods focus primarily on shallow global and local features, which may overlook important person details.

For each image, the global features and patch features extracted from ViT are represented as $[f_{cls};f_{p}^{1},f_{p}^{2},$$\cdots ,f_{p}^{N}]$. The embedded patches are divided into *i* groups and each group of patch embeddings is concatenated with the class token $f_{cls}$. Subsequently, each group of features is processed through shared a Transformer layer to generate local feature representations $f_{l}=\left[f_{l}^{1},f_{l}^{2},f_{l}^{3},\cdots ,f_{l}^{i}\right],f_{l}\in R^{i\times D}$. To address the issue of auxiliary models only extracting unobstructed pedestrian information, a feature interaction module is introduced to capture the relationships between global and local features, helping the network focus on the unobstructed and noise-free details within the global features.

#### 3.2.1. Global to Local Feature Interaction

The fusion of global and local features achieves effective interaction between them. Global features provide an overview of the overall information of the image, while local features focus on the detailed parts of the image. By introducing global features, local features not only rely on the information they carry but are also constrained and guided by the overall information of the entire image. This interaction mechanism enables the model to refer to and utilize the overall information when processing local details.

**Figure 3:**
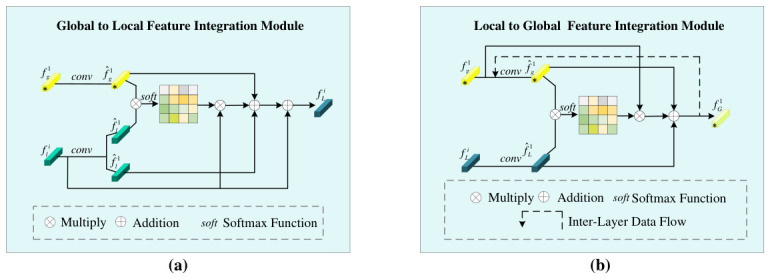
Feature Interaction Module. (**a**) Schematic diagram of Global to Local Feature Interaction module. (**b**) Schematic diagram of Local to Global Feature Interaction module.

Specifically, first, the global feature representation $f_{g}^{1}$ is extracted from the image $I_{1}$, and the local feature representations $\left[f_{l}^{1},f_{l}^{2},f_{l}^{3},\cdots ,f_{l}^{i}\right]$ are extracted from the same image, as shown in Figure 3a. Next, the global feature $f_{g}^{1}$ is input into the Global to Local Feature Interaction module to enhance the local features $f_{l}^{i}$. This process can be expressed through the following formula:

(4) $$f_{L}^{i}=GLFI\left(conv\left(f_{g}^{1}\right),conv\left(f_{l}^{i}\right)\right),i=1,2,\cdots ,n,$$

where:

- *n* represents the number of passes through the module, which equals the number of local features.
- conv(·) represents convolution operations that project features to a lower dimensional space.
- GLFI(·) denotes the Global to Local Feature Interaction strategy.
- $f_{L}^{i}\in R^{D}$ represents the enhanced local feature for the *i*-th local region.
- $f_{g}^{1}\in R^{1\times D}$ represents the global feature extracted from image $I_{1}$.
- $f_{l}^{i}\in R^{D}$ represents the original local feature for the *i*-th region.

The specific operations of GLFI(·) can be expressed by the following formula:

(5) $$f_{L}^{i}=Softmax\left({\hat{f}}_{l}^{i}⊗{\hat{f}}_{g}^{1}\right)\cdot f_{l}^{i}+{\hat{f}}_{l}^{i}+{\hat{f}}_{g}^{1}+f_{g}^{1},i=1,2,\cdots ,n,$$

where ${\hat{f}}_{g}^{1}$,${\hat{f}}_{l}^{i}\in R^{D}$ are obtained by applying $conv_{1\times 1}$ convolution operations to $f_{g}^{1}$ and $f_{l}^{i}$, respectively, and Softmax(·) denotes the Softmax function.

#### 3.2.2. Local to Global Feature Interaction

After enhancement by the GLFI module, the obtained local features have incorporated global information, emphasizing a high correlation between local and global features. However, the presence of occlusion noise still poses a challenge to the model’s recognition capability, requiring the network to focus more on those key detailed features that can accurately distinguish person identities. To address this challenge, we further integrate and interact the enhanced local features with global features, aiming to enhance the expressive power of global features so that they can more robustly reflect the unique characteristics of persons. As shown in Figure 3b, the local features in turn enhance the global features by incorporating the enhanced local features $f_{L}^{i}$ into the global features. The expression is as follows:

(6) $$f_{G_{i}}^{1}=\left\{\begin{array}{cc} LGFI(conv\left(f_{g}^{1}\right),conv\left(f_{L}^{i}\right)), & i=1,\\ LGFI(conv\left(f_{G_{\left(i-1\right)}}^{1}\right),conv\left(f_{L}^{i}\right)), & i=2,\cdots ,n, \end{array}\right.$$

where:

- *n* represents the number of passes through the module, which equals the number of local features.
- conv(·) represents convolution operations that project features to a lower dimensional space.
- LGFI(·) denotes the Local to Global Feature Interaction strategy.
- $f_{G_{i}}^{1}\in R^{1\times D}$ represents the enhanced global feature at the *i*-th iteration.
- $f_{L}^{i}\in R^{D}$ represents the enhanced local feature from the GLFI module.
- $f_{g}^{1}\in R^{1\times D}$ represents the original global feature.
- $f_{G_{\left(i-1\right)}}^{1}\in R^{1\times D}$ represents the global feature from the previous iteration.

The specific operations of LGFI(·) can be expressed through the following formula:

(7) $$f_{G_{i}}^{1}=\left\{\begin{array}{cc} Softmax\left({\hat{f}}_{g}^{1}⊗{\hat{f}}_{L}^{i}\right)\cdot f_{g}^{1}+{\hat{f}}_{L}^{i}+{\hat{f}}_{g}^{1}+f_{g}^{1}, & i=1\\ Softmax\left({\hat{f}}_{G_{\left(i-1\right)}}^{1}⊗{\hat{f}}_{L}^{i}\right)\cdot f_{G_{\left(i-1\right)}}^{1}+{\hat{f}}_{L}^{i}+{\hat{f}}_{G_{\left(i-1\right)}}^{1}, & i=2,\cdots ,n \end{array}\right.$$

where ${\hat{f}}_{g}^{1}$, ${\hat{f}}_{L}^{i}$ and $f_{G_{\left(i-1\right)}}^{1}\in R^{D}$ are obtained by applying $conv_{1\times 1}$ convolution operations to $f_{g}^{1}$, $f_{L}^{i}$ and $f_{G_{\left(i-1\right)}}^{1}$, respectively, and Softmax(·) denotes the Softmax function. By adding $f_{g}^{1}$ to $f_{G}^{1}$, the fused global feature is obtained as:

(8) $$f_{G}=\left(1-\alpha \right)f_{g}^{1}+\alpha f_{G}^{1},$$

where α is a hyperparameter.

#### 3.2.3. Loss Function

To optimize the measurement objectives and learn more stable feature representations, we adopt the triplet loss [1] to describe the similarity and difference between person features. Additionally, we choose the identity loss [25] to reduce the distance between the model’s outputs and the true labels. The learning of all global and local features is conducted under constraint conditions. The model is trained using the following loss:

(9) $$\begin{array}{cc} \; & L_{g}=\frac{1}{3}\sum_{j=1}^{3}\left(L_{id}\left(f_{g}^{j},y\right)+L_{tri}\left(f_{g}^{j},y\right)\right)\\ \; & =\frac{1}{3}\sum_{j=1}^{3}\left(-\log\left(P\left(y|W_{g}f_{g}^{j}\right)\right)+{\left|d_{\left(f_{g}^{j}\cdot f_{gp}^{j}\right)}-d_{\left(f_{g}^{j}\cdot f_{gn}^{j}\right)}+\eta \right|}_{+}\right) \end{array}$$

(10) $$\begin{array}{cc} \; & L_{L}=\frac{1}{n}\sum_{i=1}^{n}\left(L_{id}\left(f_{L}^{i},y\right)+L_{tri}\left(f_{L}^{i},y\right)\right)\\ \; & =\frac{1}{n}\sum_{i=1}^{n}\left(-\log\left(P\left(y|W_{L}f_{L}^{i}\right)\right)+{\left|d_{\left(f_{L}^{i}\cdot f_{\mathrm{Lp}}^{i}\right)}-d_{\left(f_{L}^{i}\cdot f_{\mathrm{Ln}}^{i}\right)}+\eta \right|}_{+}\right) \end{array}$$

(11) $$L_{main}=L_{G}+L_{g}+L_{L},$$

where:

- $L_{id}\left(\cdot ,\cdot \right)$ represents the identity loss function.
- $L_{tri}\left(\cdot ,\cdot \right)$ represents the triplet loss function.
- *y* is the true label of the person image.
- P(·) is the probability prediction of the classifier.
- $W_{G}$, $W_{g}$ and $W_{L}$ are the predictive classifiers for $f_{G}$, $f_{g}^{j}$ and $f_{L}^{i}$, respectively.
- $d_{\left(f_{G}\cdot f_{\mathrm{Gp}}\right)}$, $d_{\left(f_{g}^{j}\cdot f_{gp}^{j}\right)}$ and $d_{\left(f_{L}^{i}\cdot f_{\mathrm{Lp}}^{i}\right)}$ represent the distance between features of positive sample pairs (same identity).
- $d_{\left(f_{G}\cdot f_{\mathrm{Gn}}\right)}$, $d_{\left(f_{g}^{j}\cdot f_{gn}^{j}\right)}$ and $d_{\left(f_{L}^{i}\cdot f_{\mathrm{Ln}}^{i}\right)}$ represent the distance between features of negative sample pairs (different identities).
- η is the margin parameter that controls the separation between positive and negative pairs in triplet loss.
- $L_{G}$ is the loss computed on the fused global feature $f_{G}$.
- $L_{g}$ is the average loss computed on the three branch global features $f_{g}^{j}$.
- $L_{L}$ is the average loss computed on the *n* local features $f_{L}^{i}$.

### 3.3. Feature Reconstruction Module

**Figure 4:**
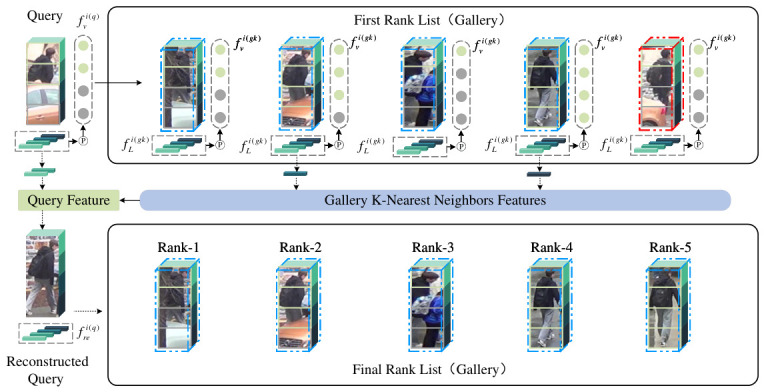
Feature Reconstruction Module. The blue dashed boxes represent correctly retrieved sample images, while the red dashed boxes indicate incorrectly retrieved ones. The first round of rank list shows the *K*-nearest similar gallery images retrieved. The final rank list presents the retrieval results after the reconstruction of the query image. The first round of rank list results are used to reconstruct the occluded parts of the query image.

In previous studies on occluded person re-identification, little attention has been paid to the impact of the OTP and the PTP on the recognition of occluded persons. The presence of the PTP not only disrupts the integrity of the person but also contaminates the true feature representation in the feature space. To reduce the interference of noise, we utilize the visible key features of complete persons in the gallery to reconstruct the features of occluded persons in the query image, as shown specifically in Figure 4.

#### 3.3.1. Prior Knowledge-Based Occlusion Simulation

A powerful data augmentation strategy, namely Prior Knowledge-based Occlusion Simulation (PKOS) [24], is introduced to address the issue of feature loss caused by occlusion in person re-identification. Specifically, almost all occluded images in the dataset have occlusions located in the lower half of the image. Based on this prior knowledge, the upper half of a randomly selected image from the sequence is used as the occlusion for the lower half of the current image. This method can effectively simulate two scenarios of occlusion: by objects or by other persons. PKOS is concatenate with the base image to form the main branch input, while setting up two additional branch inputs to augment the data and enable complementary learning, thereby ensuring that the person’s features are not completely lost. At the same time, an occlusion label is set $l_{v}$ for the replaced part to indicate whether occlusion simulation has been applied to that part’s features, with specific operations as follows:

(12) $$l_{v}=\{\begin{array}{cc} 0 & I_{PKOS}^{occ}\\ 1 & I_{PKOS}^{unocc} \end{array}\;,$$

where $I_{PKOS}^{occ}$ represents the person part simulated as occlusion in the PKOS input branch, and $I_{PKOS}^{unocc}$ represents the original visible part of the person that has not been simulated as occlusion. The $I_{PKOS}$ is input into a submodule with fully connected layers and softmax to predict the visibility score for each part of $I_{PKOS}$. The formula is expressed as follows:

(13) $$f_{v}^{i}=\mathrm{SoftMax}\left(W_{fc}\left(I_{PKOS}^{i}\right)\right),$$

where $W_{fc}$ represents the weights of the fully connected layer. And a visibility loss is set up:

(14) $$L_{v}=\frac{1}{2B}\sum_{b=1}^{2B}\left(-\log\left(P\left(l_{v}|W_{v}f_{v}^{i}\right)\right)\right),$$

where $L_{v}$ is used for training, $l_{v}$ is the visibility label, $W_{v}$ is the classifier for $f_{v}$, and 2B represents the number of images after concatenation.

#### 3.3.2. Feature Reconstruction

After obtaining the visibility scores of the person, holistic fully visible person images are selected from the initial retrieval using the ranking list to reconstruct the occluded parts of the query image. Specifically, while retaining the unoccluded local features of the query image, the unoccluded parts of the *k*-nearest holistic gallery person images retrieved in the initial search are used to reconstruct the occluded features of the query image through a visibility-weighted averaging method. The selection of parameter *k* will be discussed. The reconstructed local features are expressed as:

(15) $$f_{re}=f_{v}^{i(q)}\cdot f_{L}^{i(q)}+\left(1-f_{v}^{i(q)}\right)\cdot \frac{\sum_{k=1}^{K}f_{v}^{i(gk)}\cdot f_{L}^{i(gk)}}{\sum_{k=1}^{K}f_{v}^{i(gk)}},$$

where $f_{v}^{i(q)}$ and $f_{L}^{i(q)}$ represent the visibility score and local feature, respectively, for the *i*-th part of the person image in the query set. Similarly, $f_{v}^{i(gk)}$ and $f_{L}^{i(gk)}$ represent the visibility score and local feature, respectively, for the corresponding part in the k-th ranked list from the gallery. After feature reconstruction, the visibility scores of the query image parts are updated, and the distances between the complete query image and the gallery images used for retrieval are recalculated, which can be expressed as:

(16) $$\begin{array}{c} \mathrm{dist}\left(f_{\mathrm{re}}^{i},f_{L}^{i(gk)}\right)=\frac{\sum_{i=1}^{n}\left(f_{v}^{i(q)}\cdot \left[f_{v}^{i(q)}+\left(1-f_{v}^{i(q)}\right)\cdot \underset{i}{\arg\;\max}f_{v}^{i(gk)}\right]\cdot {∥f_{\mathrm{re}}^{i(q)}-f_{L}^{i(g)}∥}_{2}\right)}{\sum_{i=1}^{n}\left(f_{v}^{i(q)}\cdot \left[f_{v}^{i(q)}+\left(1-f_{v}^{i(q)}\right)\cdot \underset{i}{\arg\max}f_{v}^{i(gk)}\right]\right)} \end{array}$$

where dist(·,·) denotes the Euclidean distance, *n* is the number of local features, argmax indicates finding the maximum value, and ∥·∥ represents the L2 norm.

The total training loss can be formulated as:

(17) $$L_{total}=L_{main}+L_{v}.$$

## 4. Experiment

In this section, we validate the effectiveness of our proposed method through comprehensive experimental evaluations. Specifically, as shown in Table 1, we conduct experiments on seven datasets. Three of these datasets focus on occlusion scenarios: Occluded-DukeMTMC [4], Occluded-reID [26], and Partial-REID [27]. Additionally, two partial occlusion datasets are used: P-DukeMTMC-reID [26] and P-ETHZ [26]. For a more comprehensive evaluation, two holistic datasets are included: Market-1501 [28] and Duke MTMC-reID [29]. Through these diverse datasets, we aim to thoroughly assess the performance of our method under occlusion, partial occlusion, and holistic conditions.

Occluded-DukeMTMC [4] dataset is currently the largest dataset for occluded person re-identification, posing significant challenges due to its inclusion of numerous occluded images. It consists of 15,681 training images (covering 702 identities), 2210 query images (involving 519 identities), and 17,661 test images (containing 1110 identities). These images are selected from the DukeMTMC-reID [29] dataset, with occlusions introduced to simulate real-world scenarios.

Occluded-reID [26] dataset is a relatively small dataset captured through mobile phone photography, containing images of 200 identities. Each identity is characterized by 10 images, including 5 images with varying degrees of occlusion and 5 holistic images.

P-DukeMTMC-reID [26], like Occluded-DukeMTMC, is selected from the DukeMTMC-reID dataset. The P-DukeMTMC-reID training set contains 12,927 images of 665 identities, the query set consists of 2163 images from 634 identities, and the gallery includes 9053 images of 634 identities. Notably, all holistic images were removed from the query set, and all occluded person images were removed from the gallery.

P-ETHZ [26] dataset is a modified version of the ETHZ [30] dataset, containing 3897 images across 85 identities, with varying numbers of images for each identity, including both holistic and occluded views. Consistent with the methods of Jia et al. [31] and Zhuo et al. [26], we randomly select images from half of the identities in the dataset to form the training set, with the remaining identities for testing.

Partial-REID [27] dataset is designed for partial re-identification, with images covering various perspectives, backgrounds, and occlusion types. It consists of 600 images from 60 identities, each identity having 5 full-body images and 5 partially occluded images.

Market-1501 [28] is a widely used holistic person dataset, consisting of 1501 identities captured by six cameras in front of a supermarket. The dataset is divided into two subsets: a training set with 12,936 images of 751 identities and a test set with 19,732 images of 750 identities. During testing, 3368 query images are selected from the test set to retrieve corresponding individuals from the gallery.

DukeMTMC-reID [29] dataset contains 36,411 images of 1404 identities, split into 16,522 training images, 17,661 gallery images, and 2228 query images.

In order to comprehensively evaluate the performance of various person re-identification models and existing methods, this paper uses Cumulative Matching Characteristic (CMC) curves and mean Average Precision (mAP) as evaluation metrics. All experiments are conducted under a single-query setting.

### 4.1. Implementation Details

The ViT-base [15] is employed as the backbone network, pre-trained on the ImageNet dataset [32]. In our experimental configuration, all input images are resized to a consistent size of 256 × 128. A batch size of 64 is chosen, with each batch comprising 8 distinct identities, and each identity represented by 8 images. We use the Stochastic Gradient Descent (SGD) optimizer with a learning rate of 0.002, momentum of 0.9, and a weight decay of 1 × 10^−8^ for optimization. The model is trained for 100 epochs, with hyperparameters set as α = 0.7 and *k* = 5.

To ensure a fair comparison with existing methods, we adopted a consistent testing protocol for datasets lacking a specified training set. Specifically, for the Occluded-reID and Partial-ReID datasets, the training set of Market-1501 was used. For the P-ETHZ dataset, following the approach of Jia et al. [31] and Zhuo et al. [26], we randomly selected images from half of the identities in the dataset to form the training set, with the remaining images used for testing.

To investigate the impact of batch size on model performance, we conducted experiments with different batch sizes on the Occluded-DukeMTMC dataset. The results are shown in Table 2.

Based on our experiments, we observe that increasing the batch size from 32 to 64 generally improves performance, as larger batch sizes provide more diverse samples within each batch, which helps the model learn more robust feature representations. However, when the batch size exceeds 64 (e.g., 80 or 96), the performance slightly decreases. This can be attributed to the following reasons: (1) Memory constraints: Larger batch sizes require more GPU memory, which may lead to reduced training stability. (2) Overfitting risk: With larger batch sizes, the model may converge to sharper local minima, potentially reducing generalization capability. (3) Hard sample mining: Smaller batch sizes (with fewer images per identity) may provide more diverse hard samples within each batch, which can be beneficial for learning discriminative features. Based on our analysis, we believe that batch size 64 represents a good balance between training efficiency and model performance for our proposed method.

### 4.2. Experimental Results

This section presents the experimental results. In the experimental comparisons, the best results of previous methods are highlighted in blue, while our method is presented in **bold**.

#### 4.2.1. Results on Occluded Datasets

As shown in Table 3, the performance of our proposed method is compared with several existing approaches. These methods are categorized into three groups based on the backbone networks utilized. The first group employs Convolutional Neural Networks (CNNs) as the backbone [3,4,5,7,11,33,34,35,36,37,38]. Among CNN-based approaches, CAAO [38] achieves the best performance on the Occluded-Duke dataset; however, a notable performance gap remains when compared to our method. On the Occluded-reID dataset, PGFL-KD [37] delivers competitive results. For the Partial-REID dataset, PRE [11] and PCB [7] achieve the best Rank-1 and mAP, respectively.

The second group comprises methods that employ Transformer architectures as the backbone network [8,13,16,21,40,41,42,43,44,45]. In recent years, Transformers have gained significant attention in the person re-identification domain due to their outstanding performance. As shown in Table 3, FCFormer [45] achieves the best results at Rank-1 and mAP on the Occluded-Duke dataset. DCG [44] and FCFormer [45] exhibit strong performance, leading in Rank-1 and mAP on the Occluded-REID dataset, respectively. For the Partial-REID dataset, PAT [40] and DCG [44] deliver the best performance in terms of Rank-1 and mAP, respectively.

The third group comprises methods that hybridize Transformer and CNN architectures [12,46,47,48], leveraging the strengths of both models in person re-identification tasks. While this hybrid approach can partially mitigate the individual limitations of each architecture, it also introduces additional complexity, which increases the challenge of hyperparameter optimization. Consequently, significant research time is required to fine-tune the network for optimal performance. As shown in Table 3, for the Occluded-Duke dataset, hybrid methods such as EIE [47] and SPH [48] achieve the best performance in terms of Rank-1 and mAP, respectively. However, our method outperforms these approaches by 1.9% in both Rank-1 and mAP. On the Occluded-REID dataset, EIE [47] and SPH [48] attain the best results in terms of Rank-1 and mAP, respectively, but our method delivers the highest Rank-1 accuracy.

In the overall comparison, our method achieves 74.7% Rank-1 accuracy and 65.6% mAP on the Occluded-DukeMTMC dataset, outperforming all existing approaches and significantly surpassing the Baseline. On the Occluded-REID dataset, our method attains 86.5% Rank-1 accuracy and 80.7% mAP, outperforming existing methods in terms of Rank-1 performance. For the Partial-REID dataset, our method reaches 84.8% Rank-1 accuracy and 84.0% mAP, surpassing several previous methods in terms of mAP accuracy.

#### 4.2.2. Results on Holistic Datasets

On the holistic Market-1501 and DukeMTMC-ReID da- tasets, we compared our method with existing methods, categorizing them into two types. One type addresses holistic person re-identification [7,34,35,49,50,51,52,53,54,55], while the other focuses on occluded person re-identification [4,5,8,11,13,16,37,39,40,41,43,46]. The comparison results are shown in Table 4. Our method also demonstrates good performance on the holistic datasets.

On the Market-1501 dataset, our method achieves the best performance at mAP, surpassing methods based on holistic person re-identification by 0.5% at mAP accuracy, while performing comparably at Rank-1 accuracy. On the DukeMTMC dataset, our method’s performance is generally consistent with existing methods. Additionally, among methods focused on occluded person re-identification, our method also yields competitive results.

This achievement is attributed to our composite data augmentation strategy, which not only simulates scenarios where persons are occluded by various obstacles but also ensures the rationality of data augmentation, enabling the network to better learn rich and comprehensive local features. Meanwhile, the feature interaction module allows the network to focus on more discriminative features. The good performance on the datasets indicates that our method does not overfit and achieves excellent results in holistic scenarios.

#### 4.2.3. Results on Partial Datasets

On the P-ETHZ and P-DukeMTMC-reID partial datasets, our model was compared with existing methods. On the P-ETHZ dataset, our method substantially exceeds previous methods, as displayed in Table 5, which achieves 81.8% Rank-1 accuracy, 89.4% Rank-5 accuracy, and 93.5% Rank-10 accuracy. Notably, when compared to the MoS [31] method, our method demonstrates good performance in terms of mAP accuracy, with an improvement of 9.5%. On the P-DukeMTMC-reID dataset, our method was compared with existing methods, as shown in Table 6. CAAO [38] method achieved the best performance among existing methods on P-DukeMTMC-reID. However, our method demonstrates excellent performance in terms of mAP accuracy, reaching 82.3%.

### 4.3. Ablation Study

#### 4.3.1. Effectiveness of Proposed Modules

As shown in Table 7, ablation experiments were conducted by adding different modules to the Baseline to analyze the effectiveness of our proposed modules. The ablation experiments were performed on the Occluded-Duke dataset. The Baseline was successively enhanced by adding the CAM, the FIM, and the FRM module.

When the model was only combined with our proposed the CAM module, the Rank-1 and mAP for Index-1 reached 72.2% and 61.0%, respectively. This represents an improvement of 8.8% and 5.2% at Rank-1 and mAP compared to the Baseline. It can be seen that the CAM achieves good results in occluded person re-identification. Index-2, which only added the FIM module, achieved 70.5% Rank-1 and 61.0% mAP accurary, an increase of 7.1% and 4.1%, respectively, compared to the Baseline method. Index-3, which combines the CAM and the FIM, achieved 72.4% Rank-1 and 62.0% mAP accurary, representing an improvement of 0.2% and 1.0% at Rank-1 and mAP compared to Index-1, respectively. Index-4, which includes all modules, achieved optimal performance with 74.7% Rank-1 and 65.6% mAP, outperforming the Baseline by 11.3% at Rank-1 accuracy and 9.8% at mAP accuracy.

Overall, the results indicate that the three proposed modules are very effective for occluded person re-identification and can improve recognition performance.

#### 4.3.2. Analysis of Bidirectional Feature Fusion

To demonstrate the effectiveness of our bidirectional feature interaction strategy, we conduct ablation experiments with different fusion configurations. The results are shown in Table 8.

From the results, we observe the following: (1) Compared with the Baseline, both single-direction fusion methods can improve performance, verifying the effectiveness of feature interaction. (2) The Global-to-Local fusion performs slightly better than Local-to-Global, indicating that using global information to guide local feature learning is more important. (3) The proposed bidirectional fusion achieves the best performance, demonstrating that the two-way interaction between global and local features can complement each other and lead to better feature representations.

#### 4.3.3. Analysis of Hyperparameters

**Figure 5:**
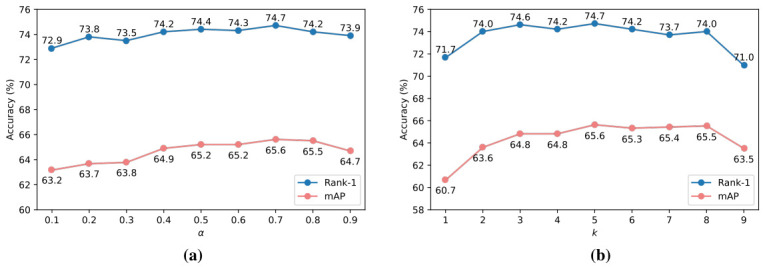
The impact of varying values of α and *k* on the performance of CAFIR is illustrated in panels (**a**) and (**b**), respectively. (**a**) A graph with different values of α and (**b**) a graph with different values of *k*.

As shown in Figure 5a, it demonstrates the impact of different α values on recognition performance. Performance tests were conducted by incrementing the value of α with a step size of 0.1 within the range of 0 to 1. The results indicate that optimal performance is achieved when the hyperparameter α is set to 0.7. In situations with varying degrees of occlusion, the model’s reliance on the initial local feature $f_{g}^{1}$ and the augmented global feature $f_{G}^{1}$ differs: the initial local feature $f_{g}^{1}$ provides some local texture details (such as distinctive backpacks and clothing), while the augmented global feature $f_{G}^{1}$ contains richer global information but may overlook some local texture features.

To find a balance under different occlusion conditions, we designed a weight α. When α is small, the model relies more on the basic local information from $f_{g}^{1}$, which is more beneficial for cases with no occlusion or mild occlusion. However, the dataset overall contains a significant number of person images with heavy occlusion. As the degree of occlusion increases, the initial local features become less informative, necessitating a higher α value to allow the augmented global feature $f_{G}^{1}$ to dominate in the fusion process.

Additionally, an excessively small α value can introduce a significant amount of noise, while an excessively large α value can result in the loss of some texture detail features. Therefore, by appropriately adjusting the α value, the model can find the optimal feature fusion method under different occlusion conditions, thereby improving the accuracy and robustness of recognition.

As shown in Figure 5b, different *k* values affect recognition performance. Notably, when k=5, both Rank-1 and mAP reach peak values, indicating that k=5 is the optimal setting for this parameter. When retrieving persons from the gallery, the ranking list inevitably contains some negative samples, which are unreliable for feature reconstruction. Therefore, to retrieve as many positive sample images as possible, the parameter *k* is set to control the number of selected samples. When *k* is small, the number of retrieved persons is limited, and the coverage of samples in the ranking list is narrow, resulting in a limited number of positive samples and affecting recognition performance. On the other hand, when *k* is large, although the number of retrieved positive samples increases, it also inevitably introduces more negative samples, adding noise during feature reconstruction and leading to a decrease in recognition accuracy. Therefore, an optimal balance between the number of positive and negative samples is achieved by setting k=5, resulting in optimal performance in the occluded person re-identification task.

#### 4.3.4. Analysis of the Number of Occluded Person Images

To investigate the impact of the number of occluded images on model performance, a series of experiments are conducted in this section. As shown in Table 9, the experimental results show that increasing the number of occluded images by 1/8 times leads to a performance improvement. As the number of occluded images continues to increase, the model performance keeps improving. These results indicate that appropriately increasing the number of occluded images can enhance the model’s robustness to occlusion and improve its generalization ability.

#### 4.3.5. Analysis of Increasing Different Types of Occluded Images

As shown in Table 10, NONE indicates no data augmentation was applied; OTP refers to generating only occluded images where pedestrians are blocked by objects in the composite data augmentation module; PTP refers to generating only occluded images where pedestrians are occluded by other pedestrians. BOTH refers to the simultaneous mixed generation of occluded images under OTP and PTP occlusion scenarios. The experimental results show that both methods can improve the model’s performance. When occluded images of both types are generated simultaneously, the model achieves the best performance, indicating that a rich and diverse training dataset helps the model better adapt to various occlusion scenarios.

#### 4.3.6. Analysis of Different Augmentation Combinations

To justify our three-branch augmentation strategy, we conduct experiments comparing different combinations of augmented inputs. The results are shown in Table 11.

The results demonstrate that our proposed three-branch combination outperforms other strategies. This verifies the effectiveness of our carefully designed augmentation strategy that pairs different augmented inputs to form triplets.

#### 4.3.7. Quantitative Evidence of Data Imbalance Alleviation

To provide quantitative evidence that our augmentation strategy alleviates data imbalance, we show the proportion of occluded samples in the training set before and after augmentation in Table 12.

As shown in the table, our augmentation strategy significantly increases the proportion of occluded samples, especially for Occluded-DukeMTMC, where it rises from 9.2% to 48.7%. This demonstrates that our method effectively addresses the data imbalance issue between training and testing sets.

#### 4.3.8. Computational Cost Analysis

To analyze the computational cost of our method, we compare it with other state-of-the-art methods in terms of model size, FLOPs, and inference speed. The results are shown in Table 13.

We observe the following: (1) Our full model has slightly higher computational cost than TransReID due to the feature reconstruction module. (2) The feature reconstruction module is optional and can be disabled for faster inference. (3) The performance degradation without FRM is relatively small (Rank-1 from 74.7% to 71.5%). This provides flexibility for users to balance between accuracy and efficiency.

#### 4.3.9. Visualization

**Figure 6:**
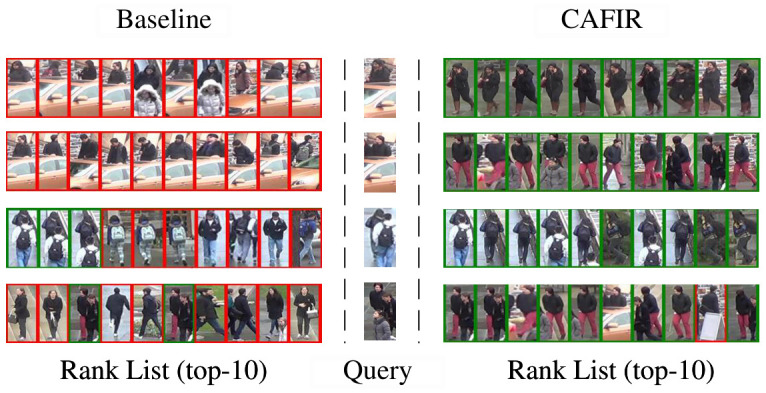
Comparison of retrieval results between Baseline and CAFIR methods. The green and red boxes denote positive and negative retrieved images, respectively.

As illustrated in Figure 6, the figure compares the actual performance of the Baseline model and our proposed method in person re-identification. Specifically, it presents the top 10 retrieval results (Rank list) for each query image under both models. In these results, green boxes indicate correctly identified positive samples (images of the same individual as the query), while red boxes denote misidentified negative samples (images of different individuals).

From the retrieval results of the Baseline model on the left, it is evident that there is a substantial presence of red boxes, which highlights the model’s tendency to generate numerous false negatives during the identification process. Particularly in occlusion scenarios, the Baseline model struggles to accurately identify positive samples that match the query image. Furthermore, when individuals exhibit similar appearances and lack distinct local features, the Baseline model’s performance in correctly identifying the target person becomes nearly ineffective.

In contrast, the retrieval results presented on the right, using our proposed method, show a marked improvement. The top 10 retrieval results under identical query conditions contain more green boxes, indicating that our method retrieves a greater number of positive samples and significantly enhances recognition accuracy compared to the Baseline model. These results demonstrate that our method excels at handling occluded person re-identification tasks and can more reliably identify individuals that match the query image.

Overall, our method exhibits a distinct advantage in occluded person re-identification tasks, substantially improving recognition performance and outperforming the Baseline model in terms of overall effectiveness.

**Figure 7:**
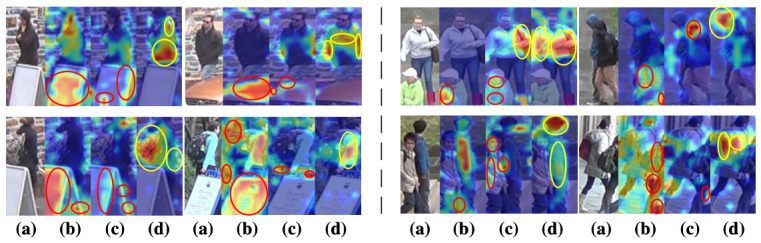
The visualization shows occluded person images with two types of occlusions: OTP (**left**) and PTP (**right**). (**a**) Input images, (**b**) Baseline heatmap, (**c**) Heatmap with composite data augmentation, and (**d**) Heatmap with both composite data augmentation and feature interaction modules. Red circles highlight noise, and yellow circles indicate person features, showing their impact on feature extraction.

As shown in Figure 7, the heatmap visualization results of occluded person images in two different scenarios are presented. The person images on the left of the dashed line correspond to the OTP scenario, while those on the right correspond to the PTP scenario. In the Baseline model, noise information is extracted during the process of person feature extraction. The presence of this noise can interfere with the model’s ability to accurately capture and recognize person features, as shown in column (b) of Figure 7.

Upon the introduction of the composite data augmentation module, the network, trained on a substantial amount of augmented data, gradually learns to disregard the occlusion-induced noise, as shown in column (c) of Figure 7. This enhancement enables the network to focus more effectively on extracting relevant person features.

Moreover, when both the composite data augmentation module and the feature interaction module are incorporated, the interaction between these two modules allows the network to not only ignore occlusion noise but also to emphasize finer, more distinguishable key features of individuals, as illustrated in column (d) of Figure 7. This precise capture of key information and effective separation of noise further enhance the recognition accuracy and robustness of the model.

## 5. Conclusions

This paper has proposed an innovative network architecture, CAFIR, to address the occluded person re-identification (re-id) problem. The architecture has leveraged the CAM to effectively increase the quantity and diversity of person image datasets under varying occlusion conditions. Additionally, the CAFIR has incorporated the FIM, which has played a critical role in capturing and distinguishing key details within person features. In the FRM, the network has utilized these extracted details to select complete images from the gallery, enabling the reconstruction of occluded body parts. To comprehensively evaluate the performance of the CAFIR, we have conducted extensive experiments on three occlusion datasets, two partial datasets, and two holistic datasets. The results have demonstrated that the CAFIR has significantly outperformed previous methods, achieving superior accuracy in person re-identification under occlusion conditions while excelling in handling non-occluded data.

Despite the CAFIR’s remarkable achievements in occluded person re-id, there has remained room for improvement in designing lighter and more efficient models to address deployment challenges on low-resource devices. Future research will focus on exploring lightweight techniques to develop a streamlined and efficient CAFIR model, aiming to reduce computational load and training time while meeting the diverse needs of practical applications.

## Figures and Tables

**Table 1:** Statistics of the seven datasets used in the experiment.

Datasets	Train		Test			
	IDs	Images	Query		Gallery	
			IDs	Images	IDs	Images
Market-1501	751	12,936	750	3368	750	19,732
DukeMTMC	702	16,522	702	2228	702	17,661
Occluded-DukeMTMC	702	15,618	519	2210	1110	17,661
Occluded-reID	-	-	200	1000	200	1000
P-ETHZ	-	-	85	546	85	3351
Partial-REID	-	-	60	300	60	300
P-DukeMTMC-reID	665	12,927	634	2163	634	9053

**Table 2:** Performance with different batch sizes on Occluded-DukeMTMC dataset.

Batch Size	Rank-1	mAP
32	73.1	63.8
48	73.9	64.5
64	**74.7 **	**65.6**
80	74.3	64.9
96	73.8	64.2

**Table 3:** Compared the performance of CAFIR with existing methods on three datasets that specifically address occlusion scenarios: Occluded-DukeMTMC, Occluded-REID, and Partial-REID.

Backbone	Method	Occluded-Duke		Occluded-REID		Partial-REID	
		Rank-1	mAP	Rank-1	mAP	Rank-1	mAP
CNN	Part-Aligned [33]	28.8	20.2	-	-	-	-
	PCB [7]	42.6	33.7	41.3	38.9	66.3	63.8
	Part Bilinear [34]	36.9	-	-	-	-	-
	FD-GAN [35]	40.8	-	-	-	-	-
	Ad-Occluded [36]	44.5	32.2	-	-	-	-
	PGFA [4]	51.4	37.3	-	-	69.0	61.5
	PVPM [3]	47.0	37.7	66.8	59.5	-	-
	HOReID [5]	55.1	43.8	80.3	70.2	85.3	-
	PGFA-KD [37]	63.0	54.1	80.7	70.3	85.1	-
	RTGAT [39]	61.0	50.1	71.8	51.0	-	-
	PRE [11]	67.1	54.3	-	-	86.0	-
	CAAO [38]	68.5	59.5	-	-	-	-
Transforme	PAT [40]	64.5	53.6	81.6	72.1	88.0	-
	OAMN [41]	62.6	46.1	-	-	86.0	77.4
	TransReID [16]	64.2	55.7	70.2	67.3	71.3	68.6
	MSDPA [42]	70.4	61.7	81.9	77.5	86.3	-
	AAformer [43]	67.0	58.2	-	-	-	-
	FED [13]	68.1	56.4	86.3	79.3	83.1	80.5
	PFD [8]	69.5	61.8	81.5	83.0	-	-
	DCG [44]	70.5	61.4	86.4	81.8	85.0	82.0
	FCFormer [45]	73.0	63.1	83.6	85.7	-	-
Mix	Pirt [46]	60.0	50.9	-	-	-	-
	DRL-Net [12]	65.8	53.9	-	-	-	-
	EIE [47]	72.8	62.7	85.7	79.9	-	-
	SPH [48]	71.9	63.7	82.6	82.1	-	-
	VitBaseline [16]	60.5	53.1	81.2	76.7	73.3	74.0
	OURS	**74.7**	**65.6**	**86.5**	**80.7**	**84.8**	**84.0**

**Table 4:** Compared the performance of CAFIR with existing methods on the two holistic datasets, Market-1501 and DukeMTMC-reID.

Types	Methods	Market-1501		DukeMTMC-reID	
		Rank-1	mAP	Rank-1	mAP
Holistic Methods	PCB [7]	92.3	77.4	81.8	66.1
	BOT [49]	94.1	85.7	76.4	66.1
	Part Bilinear [33]	90.2	76.0	82.1	64.2
	FD-GAN [35]	90.5	77.7	80.0	64.5
	OSNet [50]	94.8	84.9	88.6	73.5
	MGN [51]	95.7	86.9	88.7	78.4
	SCSN [52]	95.7	88.5	91.0	79.0
	RGA-SC [53]	95.8	88.1	86.1	74.9
	AGW [54]	95.1	87.8	89.0	79.6
	DCAL [55]	94.7	87.5	89.0	80.1
Occluded Methods	HOReID [5]	91.0	85.3	86.4	72.6
	PGFA [4]	91.2	76.8	82.6	65.5
	PGFL-KD [37]	95.3	87.2	89.6	79.5
	PAT [40]	95.4	88.0	88.8	78.2
	Pirt [46]	94.1	86.3	88.9	77.6
	OAMN [41]	93.2	79.8	86.3	72.6
	VitBaseline [16]	94.7	86.8	88.8	79.3
	TransReID [16]	95.2	88.9	90.7	82.0
	AAformer [43]	95.4	88.0	90.1	80.9
	FED [13]	95.0	86.3	89.4	78.0
	PFD [8]	95.5	89.6	90.6	82.2
	RTGAT [39]	95.3	88.2	89.1	80.2
	PRE [11]	95.3	86.5	89.3	77.8
	OURS	**95.4**	**89.0**	**90.1**	**81.0**

**Table 5:** Compared the performance of CAFIR with existing methods on the P-ETHZ partial dataset.

Methods	P-ETHZ			
	Rank-1	Rank-5	Rank-10	mAP
XQDA [56]	44.9	70.8	83.6	-
KCVDCA [57]	39.4	69.7	83.5	-
GOG [58]	49.1	79.2	90.2	-
Null Space [59]	40.1	71.5	84.4	-
DGD [60]	51.2	81.0	91.1	-
SVDNet [61]	52.2	78.8	87.4	-
REDA [2]	54.4	79.9	90.3	-
AFPB [26]	58.1	84.6	92.1	-
MoS [31]	79.5	85.7	-	66.8
OURS	**81.8**	**89.4**	**93.5**	**76.3**

**Table 6:** Compared the performance of CAFIR with existing methods on the P-DukeMTMC-reID partial datasets.

Methods	P-DukeMTMC-reID			
	Rank-1	Rank-5	Rank-10	mAP
RE [2]	83.4	-	-	67.7
PGFA [4]	69.0	-	-	61.5
DSR [62]	73.7	-	-	68.0
PCB [7]	79.4	87.1	90.0	63.9
FRR [63]	81.0	-	-	76.6
PFGL [37]	81.1	-	-	64.2
PVPM [3]	85.1	91.3	93.3	69.9
RTGAT [39]	85.6	91.5	93.4	74.3
QPM [64]	90.7	94.4	95.9	75.3
CAAO [38]	92.5	-	-	81.4
FCFormer [45]	91.5	-	-	80.7
MVI2P [65]	91.9	94.4	95.3	80.9
OURS	**91.6**	**94.5**	**95.5**	**82.3**

**Table 7:** Ablation experiment results. Performance analysis of each component in CAFIR.

Index	Methods			Accuracy			
	CAM	FIM	FRM	Rank-1	Rank-5	Rank-10	mAP
0				63.4	78.3	83.7	55.8
1	√			72.2	84.6	87.7	61.0
2		√		70.5	82.7	87.2	59.9
3	√	√		72.4	84.4	88.7	62.0
4	√	√	√	**74.7**	**85.2**	**89.1**	**65.6**

**Table 8:** Performance with different fusion strategies on Occluded-DukeMTMC dataset.

Fusion Strategy	Rank-1	mAP
Baseline (no fusion)	63.4	55.8
Global to Local only	68.9	59.8
Local to Global only	67.5	58.2
Bidirectional (proposed)	**74.7**	**65.6**

**Table 9:** Performance with different amounts of augmented images.

Amount of Augmented Images	Rank-1	Rank-5	Rank-10	mAP
1/8	72.6	83.7	87.6	61.1
1/4	72.9	84.0	88.1	61.2
1/2	73.8	85.2	88.6	64.7
1	**74.7**	**85.2**	**89.1**	**65.6**

**Table 10:** Performance with different types of augmented images.

Type of Augmented Images	Rank-1	Rank-5	Rank-10	mAP
NONE	63.4	78.3	83.7	55.8
OTP	65.5	80.1	84.9	57.0
PTP	71.8	84.0	87.6	58.9
BOTH	**72.2**	**84.6**	**87.7**	**61.0**

**Table 11:** Performance with different augmentation combinations on Occluded-DukeMTMC dataset.

Augmentation Combination	Rank-1	mAP
Baseline (no augmentation)	63.4	55.8
Only $I_{1}$ (base + PKOS)	67.5	58.2
Only $I_{1}$ and $I_{2}$	70.8	61.5
All three branches (proposed)	**74.7**	**65.6**
Alternative combination 1	71.2	61.9
Alternative combination 2	72.4	63.1

**Table 12:** Proportion of occluded samples before and after augmentation.

Dataset	Original (Training)	After Augmentation
Occluded-DukeMTMC	9.2%	48.7%
Occluded-reID	50.0%	68.3%
Partial-REID	50.0%	69.1%

**Table 13:** Computational cost comparison on Occluded-DukeMTMC dataset.

Method	Parameters (M)	FLOPs (G)	FPS
TransReID	34.2	8.7	124.5
PFD [8]	41.8	10.2	98.3
FCFormer	45.6	12.4	87.2
CAFIR (w/o FRM)	36.5	9.8	112.8
CAFIR (full)	38.2	11.5	72.4

## Data Availability

The data used in this study are available from the corresponding author upon reasonable request.
